# Substance use in rural trauma patients admitted for motor vehicle injuries before and during the COVID-19 pandemic

**DOI:** 10.1186/s40621-023-00415-y

**Published:** 2023-01-17

**Authors:** Toni Marie Rudisill, Lucie Steinmetz, James M. Bardes

**Affiliations:** 1 Department of Epidemiology and Biostatistics, West Virginia University School of Public Health, One Medical Center Drive, PO Box 9190, Morgantown, WV 26506 USA; 2 Department of Exercise Physiology, West Virginia University School of Medicine, Morgantown, WV USA; 3 Department of Surgery, Division of Trauma, Surgical Critical Care and Acute Care Surgery, West Virginia University School of Medicine, Morgantown, WV USA; 4 Department of Emergency Medicine, Division of Pre-Hospital Medicine, West Virginia University School of Medicine, Morgantown, WV USA

**Keywords:** Rural, Motor vehicle, Collision, Drugs, Alcohol, SARS-Cov2, Pandemic

## Abstract

**Background:**

Urban trauma centers reported increased substance use among individuals injured in motor vehicle collisions (MVC) after the start of the COVID-19 pandemic. Little is known about individuals admitted to rural trauma centers during this time. This study’s purpose was to describe substance use trends before and during the pandemic among individuals injured in MVC and treated at a rural Level-1 trauma center in West Virginia.

**Methods:**

A cross-sectional analysis was performed using patients’ medical records. The study population included individuals ≥ 18 years of age who received treatment for a motor vehicle-related injury between September 1, 2018, and September 30, 2021, and were tested for drugs and alcohol upon admittance. The pre-COVID-19 period was defined as September 1, 2018–March 15, 2020. The COVID-19 period was March 16, 2020–September 30, 2021. The primary dependent variable was the patients’ drug test results. The primary independent variable was the time period. The data were analyzed using Chi-square tests, logistic regression, and proportional odds models.

**Results:**

During this time, 1465 patients received treatment. On average, patients were 45 years ± 20 of age and male (57%). During COVID-19, 17% of patients tested positive for alcohol and 58% tested positive for non-alcohol drugs. After adjusting for patients’ sex and age, the number of drugs that patients tested positive for was 31% higher during COVID-19 (aOR 1.31; 95% CI 1.08, 1.58). The proportion of patients testing positive for cannabinoids (*p* = 0.05), opioids (*p* = 0.001), and stimulants (*p* = 0.010) increased from pre-COVID-19 to COVID-19 periods.

**Conclusions:**

Drug and alcohol use increased among trauma patients admitted to a rural trauma center during COVID-19. Significant increases were seen in the number of drugs and for cannabinoids, opioids, and stimulants.

## Background

Alcohol- and drug-related motor vehicle collisions remain a serious public health risk. The National Highway Traffic Safety Administration (NHTSA) has continually conducted studies using roadside data collection techniques to estimate the prevalence of drinking and drugged driving on US roadways (Lacey et al. 2009; Kelley-Baker et al. 2016) and to estimate the relative crash risk associated with drugs other than alcohol (Compton and Berning 2015; Lacey et al. 2016). During the initial response to the SARS-Cov2 (COVID-19) pandemic, our nation noted an increase in mental health diagnosis and in the use of alcohol, cannabinoids, and opioids by drivers in urban settings (Thomas et al. 2020). This is similar to prior reports that during times of increased public stress and crisis, the population reports increased rates of substance use (Bruguera et al. 2018; Vlahov et al. 2004).

Rural populations face barriers to seeking and obtaining health care and show disparities in their outcomes. In rural states, the mortality rate after motor vehicle collision can be up to four times greater than urban counterparts (Gonzalez et al. 2006). Additionally, rural populations are already known to be at increased risk of substance use as demonstrated by the opioid epidemic of the last 20 years. In particular, rural Appalachia, which includes the entire state of West Virginia, has been impacted by opioids more than any state and consistently has the highest opioid overdose death rate in the nation (Merino et al. 2019; Centers for Disease Control and Prevention 2022). Despite these known risk factors, little is known about the effect of the COVID-19 pandemic on alcohol and drug use while driving in rural areas such as West Virginia. This study was designed to investigate the effect of the COVID-19 pandemic on impaired driving in this rural state. It aimed to provide data on the frequency of both alcohol and other drugs of abuse in patients that presented to a rural trauma center after a motor vehicle collision.

## Methods

### Study design and population

**Fig. 1:**
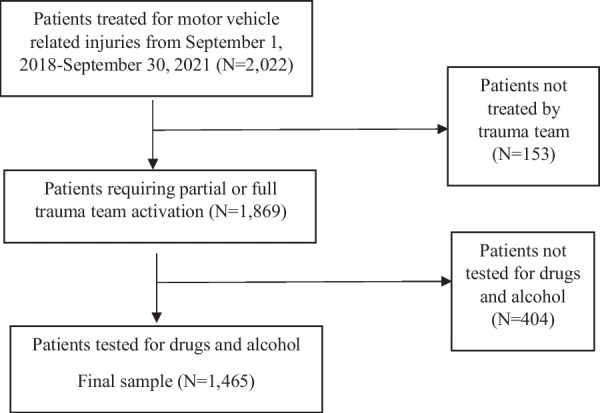
Overview of the study population included for analysis

This study was a cross-sectional analysis. The study population included any individual ≥ 18 years of age who received treatment at the Jon Michael Moore Trauma Center, Morgantown, West Virginia, for a motor vehicle-related injury where activation of the trauma team was required. The decision to activate the trauma team is based on the patient’s vital signs, mechanism of injury, and other risk factors. Eligible patients were first identified via International Classification of Disease Codes, 10th Revision, Clinical Modification, V00–V89. Each patient record was then reviewed to ensure that the individual was involved in a motor vehicle collision and subsequently received care for the incident injury; those who were not were excluded from analysis. The study population was further limited to adults for two reasons. First, drug and alcohol use patterns likely differ between adults and children (Alcover and Thompson 2020). Secondly, youth drive less than adults due to graduated drivers’ licensing laws which decrease their exposure time relative to adults (Karaca-Mandic and Ridgeway 2010). The individual had to be treated between September 1, 2018, and September 30, 2021. The study population was further limited to patients who were tested for drugs and alcohol as per trauma center protocol upon admittance. An overview of the study population is shown in Fig. 1. While the national estimates for alcohol and drug testing among trauma patients are 50% and 36%, respectively (London and Battistella 2007), 78% of patients injured in motor vehicle collisions were tested for both drugs and alcohol. The Jon Michael Moore Trauma Center is one of only two Level-I trauma centers located in the state of West Virginia.

### Variables

The primary independent variable was whether the individual was admitted before or during the COVID-19 pandemic, which is referred to as pre-COVID and COVID periods, respectively, hereafter. Because West Virginia’s Stay-at-Home order was enacted mid-March 2020, the pre-COVID period was defined as September 1, 2018–March 15, 2020. The COVID period was March 16, 2020–September 30, 2021.

The primary dependent variable in this study involved the patients’ drug test results. Patients were tested for alcohol, delta-9-tetrahydrocanabinol (i.e., THC), amphetamines, methamphetamine, 3,4-methylenedioxymethamphetamine (i.e., MDMA), cocaine, opioids in general, oxycodone, methadone, barbiturates, or benzodiazepines. Alcohol was tested via blood, whereas the other drugs were identified via urinalysis. These results were classified by the type of drug(s), the combination of drugs, and the number of drugs identified. Drug type included alcohol, cannabinoids, opioids, stimulants, and depressants. An individual was considered alcohol positive if they tested positive for any amount (i.e., ≥ 1 mg/dl). Stimulants included amphetamines, methamphetamine, MDMA, and cocaine. Opioids included oxycodone and methadone. Depressants included barbiturates and benzodiazepines. Combinations of drugs in which patients tested positive were categorized as drug positive (binary), alcohol positive (binary), drug and alcohol positive (binary). A person was considered drug positive if they tested positive for 1 or more non-alcohol drug. A person was considered drug and alcohol positive if they tested positive for 1 or more non-alcohol drugs and tested positive for any amount of alcohol (i.e., ≥ 1 mg/dl). The number of non-alcohol drugs was categorized as 0, 1, 2, or ≥ 3. Drugs received as part of pre-hospital care were excluded from analyses.

Other covariates of interest are shown in Table 1. In addition to the patient’s age and sex, their medical record was reviewed to determine whether or not they were a driver of a vehicle involved in the collision or passenger (yes/no) and whether or not they were wearing a seatbelt or not at time of the crash (yes/no). The day the patient was admitted for treatment was classified as a weekend (i.e., Saturday or Sunday) or not (i.e., Monday-Friday). The season when the individual received treatment was categorized by meteorological season in the northern hemisphere (Oceanic et al. 2022). Injury severity was coded per patients’ injury severity scores (ISS); the ISS was dichotomized as 1–15 (i.e., low to moderate severity) versus ≥ 16 (severe) (VanDerHeyden et al. 2008). The patients’ medical records were reviewed for the presence of any mental health conditions. If a patient was diagnosed with alcohol use disorder, substance use disorder, chronic drug abuse, attention-deficit hyperactivity disorder, a psychiatric illness, mental/personality disorder, or dementia, they were classified as having a mental health disorder. Patients were coded binarily (i.e., yes/no) if they had alcohol use disorder or substance use disorder/chronic drug abuse.

**Table 1:** Demographic characteristics of trauma patients tested for drugs and alcohol during pre-COVID and COVID periods^a^

Characteristic	Pre-COVID period (N = 740)		COVID period (N = 725)		Total (N = 1465)		*p*-value
	Mean	SD	Mean	SD	Mean	SD	
Age (years)	46.1	20.0	44.5	19.4	45.3	19.7	0.133
BAC (mg/dl)^b^	0.03	0.07	0.03	0.08	0.03	0.07	0.159

	N	%	N	%	N	%	
Sex							0.096
Male	406	54.9	429	59.2	835	57.0	
Female	334	45.1	296	40.8	630	43.0	
Vehicle driver							0.755
Yes	471	84.1	580	83.5	1051	83.7	
No	89	15.9	115	16.5	204	16.3	
Missing	180		30		210		
Seatbelt worn							0.626
Yes	434	71.5	459	72.7	893	72.1	
No	173	28.5	172	27.3	345	27.8	
Missing	133		94		227		
Weekend admittance							0.848
Yes	211	28.5	210	29.0	421	28.7	
No	529	71.5	515	71.0	1044	71.3	
Season of admittance							< 0.0001
December–February	216	29.2	97	13.4	313	21.4	
March–May	149	20.1	185	25.5	334	22.8	
June–August	129	17.4	263	36.3	392	26.8	
September–November	246	33.2	180	24.8	426	29.1	
Injury severity score							0.697
1–15	588	79.5	582	80.3	1170	79.9	
≥ 16	152	20.5	143	19.7	295	20.1	
Mental health condition^C^							0.683
Yes	274	37.0	261	36.0	535	36.5	
No	466	63.0	464	64.0	930	63.5	
Alcohol use disorder							0.163
Yes	40	5.4	52	7.2	92	6.3	
No	700	94.6	673	92.8	1373	93.7	
Substance use disorder/chronic drug abuse							0.001
Yes	87	11.8	130	17.9	217	14.8	
No	653	88.2	595	82.1	1248	85.2	
Alcohol positive							0.700
Yes	123	16.6	126	17.4	249	17.0	
No	617	83.4	599	82.6	1216	83.0	
Drug positive							0.072
Yes	392	53.0	418	57.7	810	55.3	
No	348	47.0	307	42.3	655	44.7	
Drug and alcohol positive							0.266
Yes	63	8.5	74	10.2	137	9.3	
No	677	91.5	651	89.8	1328	90.7	
Number of non-alcohol drugs detected							0.003
0	348	47.0	307	42.3	655	44.7	
1	250	33.8	217	29.9	467	31.9	
2	96	13.0	132	18.2	228	15.6	
≥ 3	46	6.2	69	9.5	115	7.9	

*BAC*, blood alcohol concentration; *N*, frequency; *SD*, standard deviation

^a^Pre-COVID period was defined as September 1, 2018–March 15, 2020, while COVID period was defined as March 16, 2020–September 30, 2021. Drugs exclude those that were received as part of pre-hospital care

^b^BACs presented in table are for the overall population, which included those who tested 0 mg/dl for alcohol. Among those who tested positive for any alcohol (i.e., ≥ 0.01 mg/dl), the mean (SD) for pre-COVID period was 0.17 (0.09); COVID period = 0.18 (0.08); total = 0.17 (0.09)

^C^Mental health conditions include individuals with alcohol use disorder, substance use disorder, chronic drug abuse, attention-deficit hyperactivity disorder, psychiatric illness, mental/personality disorder, or dementia

### Statistical analysis

The demographic characteristics of patients admitted to treatment were compared by pre-COVID and COVID periods via descriptive statistics (i.e., frequencies and percentages; means and standard deviations), Student’s *T*-tests (for normally distributed continuous variables), and Chi-square tests. The drug categories that patients tested positive for were compared pre-COVID and COVID periods using Chi-square tests overall and then stratified by driver status and sex. The combinations of drugs that patients tested positive for were compared pre-COVID and COVID periods using both crude and adjusted logistic regression models for binary outcomes and via proportional odds models for number of drugs, which was categorical. Models were adjusted for drivers’ sex and age as drug use is known to differ by sex and age. All data management and statistical analyses were conducted in SAS software version 9.4 (Cary, NC) with two-sided significance level *α* = 0.05.

## Results

During the study period, 1465 patients ≥ 18 years of age received treatment for motor vehicle-related injuries (Table 1). Overall, the majority of patients averaged 45 years ± 20 of age and male (57%). Overall, the majority of patients were drivers of motor vehicles (84%) and only 72% were wearing a seat belt at time of collision. The majority of crashes occurred during the weekday (71%). During COVID, a greater proportion of individuals with substance use disorder/chronic drug abuse (18%) received treatment. During COVID, 17% of admitted patients tested positive for alcohol and 58% tested positive for a non-alcohol drug.

Table 2 shows the crude and adjusted odds of patients’ drug positivity during the COVID vs. pre-COVID periods. Drug positivity and drug and alcohol positivity did not significantly differ between the COVID and pre-COVID periods for patients. However, after adjusting for patients’ sex and age, the number of drugs that patients tested positive for was 31% greater during COVID versus pre-COVID (adjusted odds ratio 1.31; 95% confidence interval 1.08, 1.58).

**Table 2:** Crude and adjusted odds of drug positivity among trauma patients comparing COVID vs. pre-COVID periods^a^

Drug category	Crude OR	95% CI	Adjusted OR^b^	95% CI
Any non-alcohol drug	1.21	0.98, 1.49	1.18	0.96, 1.45
Drug and alcohol	1.22	0.86, 1.74	1.16	0.81, 1.66
Number of drugs^b^	1.34	1.10, 1.62*	1.31	1.08, 1.58*

*CI*, Confidence interval; *OR*, odds ratio

^a^Logistic regression was run for all models except number of drugs. Proportional odds model was run for number of drugs; asterix (*) denotes *p*-value *p* ≤ 0.05

^b^Models adjusted for sex and age

When comparing specific drug categories in which patients tested positive for (Table 3), the proportion of individuals testing positive for alcohol, cannabinoids, opioids, and stimulants increased, while depressants decreased pre- COVID vs. COVID periods. The proportion of patients testing positive for cannabinoids increased from 21 to 25% from pre- to COVID periods (*p* = 0.05). The proportion of individuals testing positive for opioids (*p* = 0.001) and stimulants (*p* = 0.010) significantly increased from pre-COVID vs. COVID periods. During the COVID period, 38% of admitted patients tested positive for opioids while 18% tested positive for stimulants.

**Table 3:** Percentage of drug positivity among trauma patients during pre-COVID and COVID periods by drug category^a^

Drug category	Pre-COVID (N = 740)		COVID (N = 725)		*p*-value
	N	%	N	%	
Alcohol	123	16.6	126	17.4	0.700
Cannabinoids	154	20.8	182	25.1	0.051
Opioid	219	29.6	277	38.2	0.001
Stimulant	94	12.7	127	17.5	0.010
Depressant	117	15.8	107	14.8	0.576

^a^*p*-values obtained via Chi-square tests

When stratified by driver status (Table 4), the proportion of drivers and passengers testing positive for alcohol, cannabinoids, opioids, and stimulants increased comparing pre- vs. COVID periods. The proportion of drivers and passengers testing positive for depressants decreased from pre-COVID vs. COVID periods. However, the proportion of drivers testing positive for opioids significantly increased from 29% pre-COVID to 37% during COVID (*p* ≤ 0.05).

**Table 4:** Percentage of drug positivity among injured drivers and passengers during pre-COVID and COVID periods by drug category^a^

Drug category	Drivers (N = 1051)				Passengers (N = 204)			
	Pre-COVID (N = 471)		COVID (N = 580)		Pre-COVID (N = 89)		COVID (N = 115)	
	N	%	N	%	N	%	N	%
Alcohol	73	15.5	101	17.4	12	13.5	21	18.3
Cannabinoids	94	20.0	139	24.0	20	22.5	33	28.7
Opioid	137	29.1	214	36.9*	27	30.3	50	43.5
Stimulant	66	14.0	93	16.0	14	15.7	23	20.0
Depressant	78	16.6	89	15.3	13	14.6	12	10.4

^a^*p* values obtained via Chi-square tests. Asterix (*) denotes *p* ≤ 0.05

When stratified by sex (Table 5), the proportion of males testing positive for stimulants significantly increased from 14% in the pre-COVID period to 19% during the COVID period (*p* ≤ 0.05). For males, there were also increased proportions of those testing positive for cannabinoid and opioids, but these were not statistically significant. As for females, the proportion of those testing positive for opioids significantly increased from 28% pre-COVID-19 to 44% during COVID (*p* ≤ 0.05).

**Table 5:** Percentage of drug positivity among injured males and females pre-COVID and COVID periods by drug category^a^

Drug category	Males (N = 835)				Females (N = 630)			
	Pre-COVID (N = 406)		COVID (N = 429)		Pre-COVID (N = 334)		COVID (N = 296)	
	N	%	N	%	N	%	N	%
Alcohol	93	22.9	92	21.5	30	9.0	34	11.5
Cannabinoids	94	23.2	118	27.5	60	18.0	64	21.6
Opioid	126	31.0	148	34.5	93	27.8	129	43.6*
Stimulant	57	14.0	82	19.1*	37	11.1	45	15.2
Depressant	60	14.8	60	14.0	57	17.1	47	15.9

^a^*p* values obtained via Chi-Square tests. Asterix (*) denotes *p* ≤ 0.05

## Discussion

This study found that drug and alcohol use generally increased among patients admitted to a rural West Virginia trauma center for motor vehicle-related injuries after the COVID-19 pandemic began. The number of drugs in which patients tested positive for increased along with changes in the types of drugs used. Depressant use generally decreased over the study period while stimulant and opioid use significantly increased among treated patients; there was also a marginal increase in cannabinoid use. Opioid use increased among female patients and drivers admitted for injuries, while stimulant use significantly increased for males after the pandemic began. These findings were expected and coalesce with the extant literature.

It is common knowledge that the COVID-19 pandemic caused major disruptions to everyday life for most individuals. As the pandemic evolved, numerous public health measures, such as travel restrictions, vaccination requirements, social distancing, etc., were taken to slow the spread of the virus. These public health measures had vast social and financial implications which resulted in increased stress and poorer mental health in the US population (Chen et al. 2021; Manchia et al. Feb 2022; Nicola et al. Jun 2020). Consequently, many individuals used alcohol and other drugs to cope. Numerous studies found that drug use patterns changed as a result of the pandemic and that drug and alcohol use along with increased sales of alcohol, marijuana, and nicotine was found in the US population and beyond (Ross et al. 2022). Increased substance use is a known risk factor for trauma (Emigh et al. 2022). Thus, it is not surprising that a greater proportion of trauma patients were found positive for drugs and/or alcohol after the pandemic. While the exact reason for the slight decrease in depressants is unknown, there are some possible explanations. Other studies found that the dispensing of some chronic medications decreased during the pandemic (Clement et al. 2021). It is possible that these individuals stopped filling prescriptions, were afraid to travel to pharmacies, could not afford to fill prescriptions, or possibly substituted prescription drugs for other drugs of abuse. Additional research is needed to elucidate this.

These results shared similarities to other studies investigating the prevalence of drugs and alcohol in fatal and seriously injured road users pre-COVID and during the pandemic. The NHTSA conducted a similar study in urban Level-1 trauma centers and medical examiners offices in Charlotte, North Carolina, Jacksonville and Miami, Florida, Baltimore, Maryland, and Worcester, Massachusetts. That study found statistically significant increases in the number of drugs and increases in alcohol, cannabinoid, and opioid positivity in patients overall and for drivers specifically. The NHTSA’s study saw decreases in antidepressant use in general but not for sedatives. As for the sexes, the NHTSA’s study found increases in alcohol and cannabinoids for both males and females while increases in opioids were seen for males and stimulants for females (Thomas et al. 2020). The reverse trend was seen in West Virginia.

The slight differences in drug types seen between this study and others are explainable. West Virginia has consistently been the epicenter of the opioid epidemic and has consistently had the highest number of opioid overdoses in the nation (Centers for Disease Control and Prevention 2022). Research has shown that stimulant use is replacing opioid use in various areas throughout the USA including West Virginia (Manchikanti et al. 2022; O'Donnell et al. 2021). Thus, it is not surprising that opioid and stimulant use was higher in trauma patients treated in West Virginia vs those in urban locales. Also, while West Virginia passed a medical marijuana law in 2017, more dispensaries, processors, and growers were permitted during the pandemic which likely impacted marijuana’s availability (West Virginia Department of Health and Human Resources 2022).

The findings of this study have numerous public health implications. First, this study showed that a large proportion of road users—including drivers—were drug and alcohol positive at the time of their collision. While being positive for a drug or alcohol does not necessarily indicate impairment, it is possible that some drivers were under the influence at the time of their collision. Public health interventions to curb drug and alcohol driving may be needed in West Virginia. Secondly, it is unknown whether drug and alcohol use will continue to rise or fall as the pandemic transpires. As this study only included data through September 2021, it is unknown whether this situation worsened or improved. Future research could investigate the changes in trauma patients’ substance use in relation to the pandemic.

### Limitations

While this study adds to the extant drugged driving literature, it is not without limitation. First, the drug use reported in this manuscript may very well be an underestimate, especially concerning opioids. Drugs administered as pre-hospital care were excluded from analyses. This was done to avoid misclassifying an individual as drug positive when drug consumption may have actually occurred after the collision as part of a patient’s post-collision care (e.g., misclassification bias). Thus, in this situation, some patients may have consumed drugs such as opioids prior to collision, but also received them as post-collision care; in this scenario, these individuals would not be counted. Secondly, there are known limitations with medical records. For example, patients’ records were reviewed to determine whether they were the driver and whether they were wearing a seat belt at time of collision. Medical records were also reviewed to determine whether the patient had an existing mental health condition or substance use disorder. This information was missing from some of the patients’ records. Third, patients were only tested for a limited number of drugs. It is entirely possible that they were positive for others but were not tested for these substances. Thus, the number of drugs may be an underestimate. The study population was also limited to those tested for both drugs and alcohol which could introduce a selection bias; however, 78% of patients injured in motor vehicle crashes were tested for drugs and alcohol which is significantly higher than the national average (London and Battistella 2007). Lastly, because this study was conducted in one of only two Level-1 trauma centers located in West Virginia, the findings may not be generalizable to the entire state or other states/regions. It may be generalizable to other rural Appalachian areas. Additionally, these results may be generalizable to a rural trauma population, but may not be generalizable to the overall motor vehicle population as trauma patients tend to test positive for drugs and alcohol more than the general population.

## Conclusions

This study found that drug and alcohol use increased among trauma patients admitted to a rural Level-1 trauma center in West Virginia during the COVID-19 pandemic. Significance increases were seen in the number of drugs that patients tested positive for during the pandemic. Depressant use generally decreased over the study period while stimulant and opioid use significantly increased among treated patients; there was also a marginal increase in cannabinoid use pre-COVID versus COVID-19 periods. Public health interventions may be needed to curb drug and alcohol involved driving in this state.

## Data Availability

The datasets generated and/or analyzed during the current study are not publicly available due to existing data use agreements.

## Abbreviations

BAC: Blood alcohol concentration
COVID: SARS-Cov2
ISS: Injury severity score
MDMA: 3,4-Methylenedioxymethamphetamine
MVC: Motor vehicle collision
NHTSA: National Highway Traffic Safety Administration
OR: Odds ratio
SD: Standard deviation
THC: Delta-9-tetrahydrocanabinol
US: United States

## References

[CR1] Alcover KC, Thompson CL (2020). Patterns of mean age at drug use initiation among adolescents and emerging adults, 2004–2017. JAMA Pediatr.

[CR2] Bruguera P, Reynolds J, Gilvarry E (2018). How does economic recession affect substance use? A reality check with clients of drug treatment centres. J Ment Health Policy Econ.

[CR3] Centers for Disease Control and Prevention. Drug overdose mortality by state. https://www.cdc.gov/nchs/pressroom/sosmap/drug_poisoning_mortality/drug_poisoning.htm. Updated 1 Mar 2022. Accessed 15 Sept 2022.

[CR4] Chen J, Vullikanti A, Santos J, Venkatramanan S, Hoops S, Mortveit H (2021). Epidemiological and economic impact of COVID-19 in the US. Sci Rep.

[CR5] Clement J, Jacobi M, Greenwood BN (2021). Patient access to chronic medications during the Covid-19 pandemic: evidence from a comprehensive dataset of US insurance claims. PLoS ONE.

[CR6] Compton R, Berning A. (2015). Drug and alcohol crash risk [Traffic Safety Facts]. (Report No. DOT HS 812 117). Washington, DC: United States Department of Transportation, National Highway Traffic Safety Administration.

[CR7] Emigh B, Clark DH, Schellenberg M (2022). The impact of coronavirus 2019 on trauma. Curr Opin Anaesthesiol.

[CR8] Gonzalez R, Cummings G, Mulekar M (2006). Increased mortality in rural vehicular trauma: identifying contributing factors through data linkage. J Trauma Inj Infect Crit Care.

[CR9] Karaca-Mandic P, Ridgeway G (2010). Behavioral impact of graduated driver licensing on teenage driving risk and exposure. J Health Econ.

[CR10] Kelley-Baker T, Lacey JH, Berning A (2016). 2013–2014 national roadside survey of alcohol and drug use by drivers: methodology. (Report No. DOT HS 812 294).

[CR11] Lacey JH, Kelley-Baker T, Furr-Holden D (2009). 2007 National roadside survey of alcohol and drug use by drivers: methodology. (Report No. DOT HS 811 237).

[CR12] Lacey JH, Kelley-Baker T, Berning A (2016). Drug and alcohol crash risk: a case-control study (Report No. DOT HS 812 355).

[CR13] London JA, Battistella FD (2007). Testing for substance use in trauma patients: are we doing enough?. Arch Surg.

[CR14] Manchia M, Gathier AW, Yapici-Eser H (2022). The impact of the prolonged COVID-19 pandemic on stress resilience and mental health: a critical review across waves. Eur Neuropsychopharmacol.

[CR15] Manchikanti L, Singh VM, Staats PS (2022). Fourth wave of opioid (illicit drug) overdose deaths and diminishing access to prescription opioids and interventional techniques: cause and effect. Pain Phys.

[CR16] Merino R, Bowden N, Katamneni S, Coustasse A (2019). The opioid epidemic in West Virginia. Health Care Manag.

[CR17] Nicola M, Alsafi Z, Sohrabi C (2020). The socio-economic implications of the coronavirus pandemic (COVID-19): a review. Int J Surg.

[CR18] National Oceanic and Atmospheric Administration. Meteorological versus astronomical seasons. https://www.ncei.noaa.gov/news/meteorological-versus-astronomical-seasons. Accessed 16 Dec 2022.

[CR19] O'Donnell J, Tanz LJ, Matt Gladden R, Davis NL, Bitting J (2021). Trends in and characteristics of drug overdose deaths involving illicitly manufactured fentanyls—United States, 2019–2020. MMWR.

[CR20] Ross JA, Malone PK, Levy S (2022). The impact of the severe acute respiratory syndrome coronavirus 2 (SARS-CoV-2) pandemic on substance use in the United States. Clin Infect Dis.

[CR21] Thomas DF, Berning A, Darrah J (2020). Drug and alcohol prevalence in seriously and fatally injured road users before and during the COVID-19 public health emergency (Report No. DOT HS 813018).

[CR22] VanDerHeyden N, Cox TB, Asensio JA, Trunkey DD (2008). Chapter 6-trauma scoring. Current therapy of trauma and surgical critical care.

[CR23] Vlahov D, Galea S, Ahern J (2004). Sustained increased consumption of cigarettes, alcohol, and marijuana among manhattan residents after September 11, 2001. Am J Public Health.

[CR24] West Virginia Department of Health and Human Resources. West Virginia Medical Cannabis Making Strides in 2022. https://dhhr.wv.gov/News/2022/Pages/West-Virginia-Medical-Cannabis-Making-Strides-in-2022.aspx#:~:text=The%20Medical%20Cannabis%20Act%2C%20passed,time%20encouraging%20economic%20development%20statewide. Updated 4/21/2022. Accessed 15 Sept 2022.

